# Base of Tongue Tuberculosis: A Case Report

**Published:** 2015-05

**Authors:** Carlos Miguel Chiesa Estomba, Ana Sofia Araujo da Costa, Teresa Rivera Schmitz, Pedro Vaamonde Lago

**Affiliations:** 1 *Departmentof Otorhinolaryngology – Head & Neck, University Hospital of Vigo, Pontevedra, Spain.*; 2 *Department of Otorhinolaryngology- Head & Neck, University Hospital of Santiago de Compostela, A Coruña, Spain.*

**Keywords:** Pharynx, Tuberculosis, Tongue

## Abstract

**Introduction:**

Tuberculosis is an infectious disease that has displayed increasing incidence in the last decades. It is estimated that up to 20% of tuberculosis cases affect extra-pulmonary organs. In the ENT area, soft palate and tongue are the least probable locations.

**Case Report:**

A 62-year-old female with a history of rheumatoid arthritis and treatment with corticosteroids and Adalimumab, developed a foreign body sensation in the pharynx accompanied by a sore throat and halitosis. The laryngoscopy with a 70 degree rigid telescope showed an ulcerated hypertrophic lesion in the right vallecula of about 2-3 cm in the base of the tongue. Acid-alcohol resistant bacilli were found positive for M. tuberculosis, through the Ziehl Neelsen method and Löwenstein culture the patient was treated with tuberculostatic medication.

**Conclusion:**

TB is a possible diagnosis when in the presence of an ulcerated lesion at the base of the tongue, accompanied by sore throat, dysphagia, or foreign body sensation.

## Introduction

Tuberculosis is an infectious disease that has displayed increasing incidence in the last decades. In 2011, the WHO estimated the incidence of tuberculosis around 8.3 - 9 million cases worldwide (1). The abundance of migrations, the appearance of resistant strains, the increasing poverty, and increasing number of immune-suppressed patients are some of the reasons for such a development.

It is estimated that up to 20% of tuberculosis cases affect extra-pulmonary organs (2). In the ENT area, the most common onset is cervical lymphadenitis, which accounts for 95% of ENT cases. All other locations such as the larynx, ear, nasal passages, pharynx, tonsils, mastoid, nasopharynx, or salivary glands, account each for less than 1% of all cases(3). Especially rare is the location in the oropharyngeal region, in which the palatine tonsils are affected in 45% of the cases. The posterior pharyngeal wall, tonsillar pillars, sidewalls, soft palate, and tongue are the least probable locations (4).

To achieve a diagnosis, these lesions require cytological or histological confirmation, as they are macroscopically difficult to differentiate from other pathologies such as other granulomatous diseases or even cancer.

## Case Report

The case of a 62-year-old female with a history of rheumatoid arthritis, treatment with corticosteroids and Adalimumab, and smoking around 10 cigarettes a day, is presented The patient was admitted to the emergency service, having developed a foreign body sensation in the pharynx in the previous weeks, accompanied by a sore throat and halitosis. There was no report of fever, weight loss, or any foreign body ingestion.

**Fig1:**
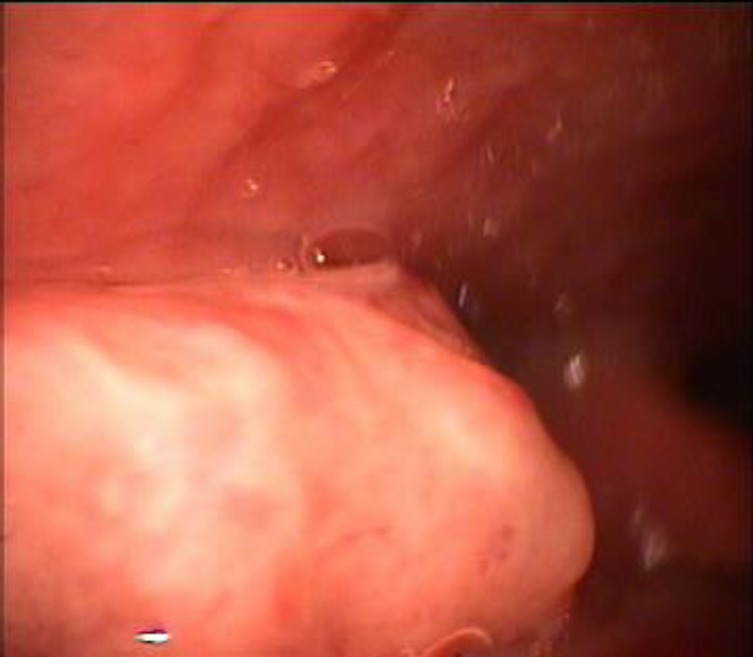
Laryngoscopy with a 70 degree rigid telescope showed an ulcerated lesion in the right vallecula of about 2-3cm based on a local hypertrophy of the base of the tongue

In the physical examination there were no evident lymph nodes in the cervical region.The laryngoscopy with a flexible naso- fibroscopic and 70 degree rigid telescope showed an ulcerated lesion in the right vallecula of about 2-3cm based on a local hypertrophy of the base of the tongue (Fig.1). The rest of the examination was without findings.

**Fig 2:**
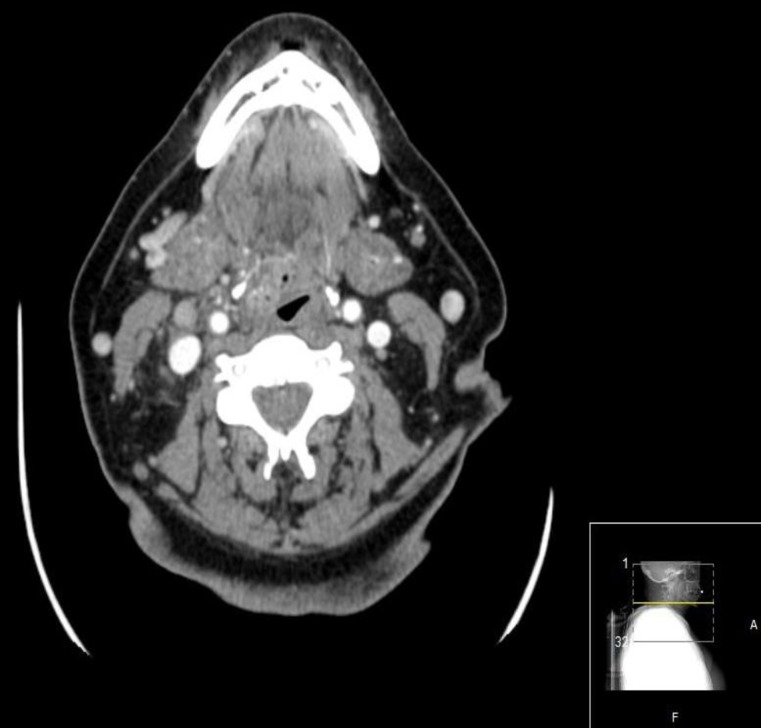
Cervical CT scan with a mass in the right vallecula contacting with the lateral posterior part of the tongue and partially obliterating the lumen of the aero-digestive way

A cervical CT scan showed a mass in the right vallecula contacting with the lateral posterior part of the tongue and partially obliterating the lumen of the aero-digestive way (Fig.2). 

The chest x-ray revealed findings suggestive of tuberculosis in the right upper lobe, which led to running a BK test of the sputum, which came out negative. A biopsy of the ulcerated lesion was performed. Histological analysis revealed focal necrotizing granulomatous inflammation; which presented acid-alcohol resistant bacilli through the Ziehl Neelsen method and was positive for M. tuberculosis by Löwenstein culture.

Being diagnosed for base of tongue tuberculosis, the patient was treated with tuberculostatic medication for 6 months; showing evident clinical improvement. 

## Discussion

Tuberculosis of the oral cavity may be primary, affecting any of its organs, or secondary to a primary lung focus, through contaminated sputum or hematogenous spread (5,6). Morgagni described the first case of lingual tuberculosis in 1761.

Lingual tuberculosis, despite being a very rare clinical condition, usually corresponds to the most frequently affected structure of the oral cavity (7), in general, and the lateral surface of the tongue, in particular (8).

Clinically, the patient usually presents a pharyngeal foreign body sensation, odynophagia, or dysphagia; and, through physical examination, a granulomatous, ulcerated and painful lesion is found (5,6). In 1971, Davis Bowen described 3 different clinical presentations of TB in the oral cavity: the acute miliary TB, the chronic ulcerative TB, and the lupus vulgaris TB. The chronic ulcerative form is the one which is usually associated with the pulmonary region (9).

The differential diagnosis of a tuberculous ulcer of the oral cavity includes aphthous ulcers, traumatic ulcers, syphilitic ulcers, actinomycosis, Wegener granuloma, and malignancy (10). The latter is more important, because tongue tumors represent one of the most frequent locations of the upper aerodigestive tract: 90% of these corresponds to epidermoid carcinomas and are associated with a high mortality rate and a high risk of metastasis.

As for treatment, chemotherapy with 4 tuberculostatics (Isoniazid, Rifampicin, Pyrazinamide and Ethambutol) is currently the most commonly used scheme and the most common method of application is the use of Isoniazid, Rifampicin, Pyrazinamide and Ethambutol for 8 weeks and then a continuation phase with Isoniazid and Rifampicin for 18 weeks (11). Once treatment begins, clinical symptoms subside in a few days while lesions improve after a few weeks (12).

## Conclusion

Despite being an uncommon condition, one should always rule out TB as a possible diagnosis when in the presence of an ulcerated lesion at the base of the tongue, accompanied by sore throat, dysphagia, or foreign body sensation. 

## References

[1] WHO (2012). Global tuberculosis report: 2012.

[2] Yang Z, Kong Y, Wilson F, Foxman B, Fowler AH, Marrs C (2004). Identification of risk factors for extrapulmonary tuberculosis. Clin Infect Dis.

[3] Nalini B, Vinayak S (2006). Tuberculosis in ear, nose, and throat practice: its presentation and diagnosis. Am J Otolaryngol.

[4] Moulonguet L, Delguidice P, Chauvin JL (1995). L’angine tuberculeuse. A propos d’un cas au Sénégal. Ann Otolaryngol Chir Cervicofac.

[5] Kakisi OK, Kechagia AS, Kakisis IK, Rafailidis PI, Falagas ME (2010). Tuberculosis of the oral cavity: a systematic review. Eur J Oral Sci.

[6] Eng HL, Lu SY, Yang CH, Chen WJ (1996). Oral tuberculosis. Oral Surg Oral Med Oral Pathol Oral Radiol Endod.

[7] Fujibayashi T, Takahashi Y, Yoneda T, Tagami Y, Kusama M (1979). Tuberculosis of the tongue. A case report with immunologic study. Oral Surg Oral Med Oral Pathol.

[8] Soni NK, Chatterjee P, Nahata SK (1980). Tuberculosis of the tongue. Ind J Tub.

[9] Bhandarker PD, Kasbekar VG, Shah RP, Hakim PP (1993). Primary tuberculous ulcer of the tongue; a case report. Tropical Doctor.

[10] Hussaini J, Mutusamy S, Omar R, Rajago- palan R, Narayanan P (2012). Base of tongue tuberculosis: a case report. Acta Med Iran.

[11] Centers for Disease Control Prevention (2003). Treatment of Tuberculosis, American Thoracic Society, CDC, and Infectious Diseases Society of America. MMWR.

[12] Jawad J, El-Zuebi F (1996). Primary lingual tuberculosis: A case report. Journal of Laryngology and Otology.

